# The Responsiveness of Mental Health Service Professionals to Two Years of Pandemic Emergency in Italy

**DOI:** 10.1007/s10488-023-01284-9

**Published:** 2023-07-10

**Authors:** Lorenza Magliano, Ginevra Di Maio, Chiara Papa, Tommaso Bonavigo, Fabrizio Starace, Gaetana Affuso

**Affiliations:** 1 Department of Psychology, University of Campania “Luigi Vanvitelli”, Viale Ellittico 31, Caserta, I-81100 Italy; 2 Department of Mental Health, Azienda Sanitaria Universitaria Giuliano Isontina (ASUGI) (Giuliano Isontino Health University District (ASUGI), Trieste, Italy; 3 Department of Mental Health and Drug Abuse, Azienda Unitá Sanitaria Locale (AUSL) Modena (Local Unit Health Agency of Modena), Modena, Italy

**Keywords:** COVID-19, Mental health staff, Mental health services, Staff views

## Abstract

This multicenter cross-sectional study explored the responsiveness of Mental Health Services (MHS) to two years of COVID-19 emergency in Italy. Specifically, the study explored the ability of staff to: acknowledge users’ capabilities and value teamwork; reinvent the service and maintain/introduce good practices; and, acknowledge the positive aspects of the pandemic experience. These aspects were investigated in relation to socio-demographic and professional variables. Professionals from 17 MHS of 15 Italian Regions completed an online questionnaire on the MHS transformation during COVID-19. Data were collected at the end of the national health emergency (March 1-April 30, 2022). Most of the 1077 participants said they: paid more attention to users’ physical health; revised treatment plans; mediated between user needs and safe work procedures; revalued the importance of gestures and habits; discovered unexpected personal resources in users; and, found positive aspects in the COVID-19 experience. The multivariate analyses showed significant differences in staff opinions related to gender, workplace, professional role, and geographic area of the MHS, covarying with staff work experience. Compared to male staff, female staff perceived MHS as more flexible and capable to maintain best practices, and female staff acknowledged more capabilities to the users. Compared to central and northern Italy staff, southern Italy staff gave more values to teamwork, perceived MHS as more capable to maintain best practices and acknowledged higher positive transformations. These findings may be useful for planning community-oriented MHS in the post-pandemic period, taking into account both the experience gained by staff and the MHS process of adaptation.

## Introduction

From the earliest stages of the pandemic, it was evident that COVID-19 was not only a serious infectious disease but also an unprecedented condition with direct and indirect effects on global mental health. Research has shown an increase of mental health problems in the general population, (Richter et al., 2021; Cuomo et al., 2022) and a worsening of clinical conditions in people with pre-pandemic mental disorders (Gillard et al., 2021; Shah et al., 2022). This situation has led to a huge burden on Mental Health Services (MHS) staff, particularly in the early pandemic phases (Carpiniello et al., 2020 and 2022; De Girolamo et al., 2020; Duden et al., 2022; Sheridan Rains et al., 2021). High levels of stress, anxiety, depression, and burnout were found among MHS staff (Foye et al., 2021; Minelli et al., 2022; Rapisarda et al., 2022), mainly related to abrupt job changes, fear of contagion, and difficulties in ensuring adequate levels of care to users and the general population (Duden et al., 2022). Studies revealed that: anxiety and emotional exhaustion were higher among female vs. male staff (Minelli et al., 2022); workload, psychological distress, and burnout were higher among inpatient vs. outpatient staff (Foye et al. 2021; Rapisarda et al., 2022); anxiety, depression and stress-related symptoms were higher among nurses, and milder among psychologists and psychiatrists vs. other professionals (Minelli et al., 2022). Moreover, burnout resulted higher among staff with shorter work experience in mental health field (Sadek et al., 2021).

Alongside the flood of studies on negative effects of COVID-19 on the MHS, few research investigated whether the pandemic had also had positive effects on MHS. Data from studies conducted in the initial stages of the pandemic showed increased staff cooperation, reduced bureaucracy, and greater flexibility of the MHS (Bommersbach et al., 2021; Guan et al., 2021; Itzhaki-Braun, 2021; Johnson et al., 2021; Pappa et al., 2021). In MHSs with adequate digital resources (i.e., internet connectivity, availability of devices for users and operators, appropriate digital literacy), remote work was evaluated as useful to facilitate participation in staff meetings and to ensure psychological support and clinical assessments for users (Di Carlo et al., 2021; Johnson et al., 2021; Sheridan Rains et al., 2021). As stated by Amerio et al. (2023), COVID-19 pandemic represented “an opportunity to overcome normative, technological, and cultural barriers to the use of online psychotherapy, showing the importance of adapting the therapeutic setting to both collective and individual needs”. Staff resilience and satisfaction for support received by health agencies were also found (Pappa et al., 2021). Among mental health staff, satisfaction with their abilities to cope with the pandemic and the knowledge that they had learned something from the experience emerged (Agrest et al., 2022). In a study on the MHS responsiveness to the pandemic carried out in England (Mannion et al., 2022), participants (managers, physicians, patient representatives and staff in charge) believed that the MHS had been able to implement new models of care and develop digital solutions, and that the MHS showed great flexibility and resilience. Another study on potential positive effects of the pandemic on the services was conducted in a single MHS in Italy one year after the onset of the pandemic (Magliano et al., 2022). In the preliminary phase of the study, an ad hoc 30-item questionnaire was developed, using a participatory methodology and taking into account the testimonies of mental health staff. The instrument addressed, from the staff perspective, pandemic-related changes in MHS practices and organization and in staff-user relationships. Data collected online revealed that, for most participants the pandemic experience had some positive aspects. Participants stated they had changed practices, reinvented the service, re-evaluated teamwork and discovered unexpected capabilities in the users.

More than two years after the start of the pandemic, it is worthwhile to begin to take stock of what this health and humanitarian emergency has represented - and in part is still representing - for MHS. It is likely that the persistence of COVID-19 has led MHS staff to develop new organizational and intervention approaches to mental health care, as to rediscover the value of teamwork and relationships with users. This may also be the case in Italy, “one of the few countries in the world where community psychiatry has been the national policy” for more than four decades (Fioritti, 2018). In Italy, the 1978 Psychiatric Reform Law enshrined that mental health care was provided to all citizens through MHS. These services, which are part of Local Health Trusts, provide treatments to the catchment area population through a network of facilities including mental health centers, day hospitals, day care centers, residential facilities and crisis management units located in general hospitals (Fioritti, 2018).

Among European countries, Italy was the first to be severely affected by the pandemic and the third in terms of the number of deaths (WHO, 2022), with higher lethality in Northern Italy particularly during the first pandemic waves (Ministero della Salute, 2022a). In the initial stages of the COVID-19, a marked North-Central-South gradient of infections was observed, while in 2021 a more widespread spatial distribution of infections was found, although the incidence remained higher in the North (ISTAT, 2022a). Mortality was particularly high in residential facilities (Istituto Superiore di Sanità, 2022), which were more concentrated and larger in northern Italy (ISTAT, 2022b). As the vaccination campaign progressed, mortality decreased significantly across the country, and as of July 2021, excess mortality in Italy fell below the EU average (ISTAT, 2022a).

On January 31, 2020, the Italian Government declared a Public Health Emergency of International Concern until March 31, 2022, without applying any specific regulations to MHS. In the early pandemic phase, main scientific societies in the field of mental health in Italy, as the Italian Society of Psychiatric Epidemiology (Starace & Ferrara, 2020) and the Italian Society of Psychiatry (SIP, 2020), released operational guidelines to support MHS in maintaining acceptable levels of care. In the first pandemic wave (February-May 2020) in MHS it occurred that: general hospital psychiatric units were in part converted into Covid-19 wards; mental health centers guaranteed limited clinical monitoring and pharmacological treatments to most severely affected users and crisis management; residential facilities remained operational but they reduced almost entirely rehabilitative activities and users’ contacts with family; day-centers have almost zeroed out in-presence rehabilitative activities and partly converted them to remote activities (Carpiniello et al., 2020; Percudani et al., 2020). Since the summer of 2020, within the limitations imposed by national guidelines to limit contagions, there was a gradual restoration of the standard of care, with significant differences among the regions (Carpiniello & Vita, 2022; Castelpietra et al., 2021; Ministero della Salute, 2022b). It is likely that the procedures put in place to face the pandemic had a significant impact on the organization of care and staffing and on the delivery of interventions at short and mid-term.

Given the paucity of information on potential pandemic-induced positive transformations in MHS, we conducted a study focused on the responsiveness of Italian MHS to two years of COVID-19 pandemic. The study was carried out in MHS of different Italian regions, and data were collected online concurrently with the end of the national pandemic state of emergency (March 31, 2022) by using the same questionnaire of the Magliano et al.2022 study. In the study presented herein, staff opinions on the following aspects were investigated: (a) recognition of user capacity; (b) awareness and value of teamwork; (c) flexibility and ability to reinvent the MHS; (d) retention and introduction of best practices; and (e) recognition of the positive aspects of the pandemic experience. Specifically, we sought to understand whether aspects a-and above varied in relation to gender, professional role, workplace, and geographic area in which the participating MHSs were located.

## Methods

### Study Design and Setting

The study was coordinated by the Department of *** of the University of *** - Italy and conducted in accordance with the Declaration of Helsinki. The study protocol was approved by the Ethics Committee of the Department of *** of the University of *** (authorization no. 1 of 2/2/2021).

The study design involved initial declaration of interest at the level of the potentially participating MHS and subsequent informed consent from individual participants. Potential participating MHS were identified through the MHS National College (Collegio Nazionale dei Dipartimenti di Salute Mentale), an association of Italian MHS Directors and Unit Coordinators (Collegio Nazionale dei Dipartimenti di Salute Mentale, 2020). On March 2022, the MHS National College President sent an invitation to the association’s members to participate in a study in MHS transformations during the pandemic (mail recipients: 42 MHS Directors and 50 Unit Coordinators). In each adhering MHS, eligible sample included health and non-health professionals working in the Adult Mental Health sector and the third sector agencies having contractual commitments with MHS (cooperatives). Twenty-three MHS who responded expressing general interest in the study topic were subsequently contacted by the study coordinating center. Of these, 17 MHS located in 15 Regions across Italy participated in the study (8 Regions in Northern Italy, 2 in Central and 5 in Southern Italy Regions) and 6 MHS did not (2 for expected poor staff participation, 2 for need of further approval by the local health agency’s ethics committee; 1 for delayed contact; 1 not reported).

### Data Collection Procedures

Data collection was conducted from March, the 1st to April, the 30th, 2022, in coincidence with the end of the national health emergency. Eligible staff received an invitation mail from the MHS Director to participate in an online study on their views regarding the MHS transformations over the two-year pandemic. Staff was also solicited to participate by WhatsApp and mail from Unit Heads and Cooperative Managers. Professionals who agreed were asked to complete online the anonymous Questionnaire on MHS Transformations during Covid-19 - staff version (QT19-S), accessible via a link contained in mails and WhatsApp messages. Participants completed the QT19-S using their personal devices. The questionnaire did not contain any mandatory response questions. Respondents could at any time abandon filling out the questionnaire by closing the Internet page. The answers already given were thus automatically deleted.

### Assessment Instrument

The QT-S is a self-reported questionnaire investigating staff opinions on potential positive transformations occurred in MHS during the pandemic. The tool was developed by a working group of MHS researchers, clinicians, and healthcare coordinators (N = 7), using a participatory research approach. On September 2020, the working group members individually analyzed six staff testimonies presented at a webinar addressing the effects of the pandemic on the MHS in Italy (details in Magliano et al., 2022). Each working group member identified prevalent themes from the video-interventions and rephrased the speakers’ statements in the form of items. The resulting 232 preliminary items were individually scored for clarity/appropriateness on a 10-point scale, from 1 “not at all” to 10 “completely,“ and for inclusion/exclusion in the instrument. Items with a clarity/appropriateness score < 7 and/or eligible for inclusion by less than 5/7 participants were removed (N = 160). The remaining 72 items were further reviewed by the participants and 25 redundant items were eliminated. The remaining 47 items were once again evaluated, and 17 more were ruled out. The final 30 items were grouped by content as follows: (a) acknowledgement of user capabilities (5 items); (b) awareness and value of teamwork (8 items); (c) flexibility and ability to reinvent the service (4 items); (d) maintenance and introduction of best practices (12 items); (e) acknowledgement of positive aspects in the pandemic experience (1 item). The rating scale was set at 6 levels, from 1 “not really true” to 6 “really true.“ To the 30 items described above, the following sections were added: two additional open-ended items on the respondent opinions on the most positive and most negative effects of the pandemic on MHS (not analyzed in this study); and a section on the respondent main sociodemographic and professional characteristics. The above-described tool was anonymously completed by professionals from the Trieste and Gorizia MHS at one-year pandemic. On the sample of 184 MHS participating staff, Confirmatory Factor Analysis was performed, applying maximum likelihood estimation of covariances. Content validity of the confirmed factors was analyzed by calculating Cronbach’s α values. Confirmatory Factor Analysis validated the five-factor structure. The final model fit the data well: χ^2 ^(396) = 900.24, p < .001; nonnormalized fit index = 0.90; comparative fit index = 0.91; root mean square error of approximation = 0.087, confidence limits 90% (0.080; 0.095); standardized root mean square residual = 0.086. All factor loadings were significant at the p < .001 level. Factor loading values ranged from: 0.33 and 0.72 for factor 1 Acknowledgement of user capabilities; from 0.43 to 0.83 for factor 2 Awareness and value of teamwork; from 0.54 to 0.70 for factor 3 Flexibility and ability to reinvent the service; from 31 to 0.72 for factor 4 Maintenance and introduction of best practices. Finally factor loadings value was fixed to 1 for factor 5 Acknowledgement of positive aspects in the pandemic experience. The correlations among the five factors were all significant for p < .001. Cronbach’s α values were: 0.68 (factor 1), 0.83 (factor 2), 0.71 (factor 3), and 0.79 (factor 4). This tool was used to collect data in the present study.

### Statistical Analysis

Descriptive statistics were computed on QT19-S items and participants’ socio-demographic (age, sex, educational level) and professional characteristics (professional role, workplace, MHS geographical location, years of working in the mental health field). Mean scores of each QT19-S a-d factors were also computed. Four Multivariate Analysis of Variance (MANOVA) with Bonferroni’s post-hoc tests were used to compare the mean scores of the five QT19-S factors (dependent variables) against each independent variable: gender, work settings (community mental health centres; day-centres; general health psychiatric units and day-hospitals; residential facilities; cooperatives), professional role (psychiatrists; psychologists; nursing staff; educators, rehabilitation staff, social workers; health assistants and other cooperative staff), MHS’s geographical area (northern Italy; central Italy; southern Italy). MANOVAs were adjusted for respondent’s working years in mental health field (covariate). The adjustment was due: (a) to interest in testing the effect, if any, of mental health work experience on the dependent variables; (b) to preliminary analyses that showed statistically significant differences in working years by respondents’ gender (F = 17.78, df 1, 1002; p< .0001), work setting (F = 8.26; df 4, 957; p < .0001), professional role (F = 13.92; df 4, 978; p < .0001), and MHS’s geographical area (F = 13.61; df 2, 954; p < .0001). When calculating the 5-factor mean scores, missing data in individual items were replaced by the mean scores of valid item responses. Missing data on participants’ socio-demographic and professional variables were not replaced, opting to perform multivariate analyses on one independent variable at a time. Statistical significance level was set at *p* < .05. Analyses were performed using the SPSS package, version 21 (IBM, 2012).

## Results

### Descriptive Results

Overall, 1077 professionals completed the online questionnaire. Participants were middle aged and mostly female and highly educated (Table 1). Most professionals were nurses, psychiatrists and rehabilitation staff and they had a rather long experience in the mental health field. Most participants worked in community mental health centers and in residential facilities. The descriptive percentages of responses to the QT-19 items are shown in Table 2. 53.7% of respondents said they discovered unexpected personal resources in users and 38.0% acknowledged that some users were more capable of using technologies than the staff themselves (factor 1, scores 5 and 6). 82.0% of respondents stated that during the pandemic they realized the importance of simply gestures and habits and 52.7% affirmed that there was more sharing of responsibility within the team (factor 2). 80.8% of participants said they were able to mediate between user demands and the procedures needed to work safely and 77.4% stated they had reinvented own way of working in line with governmental mandates (factor 3). As far as the Maintenance and introduction of best practices (factor 4), the totality of items had more than 50% of responses in score 5 and 6. Notably, 86.5% of respondents stated that the took more better care maintaining cleanliness in the work environment. 80.0% stated they had increased telephone contact with users, and 78.6% said they learned to use digital technologies better. 84.1% of respondents stated that they had informed users on procedures to reduce individual risk of contagion, 60.1% stated they had paid greater attention to users’ physical health, and 63.7% of respondents reported they had revised individual treatments plans. Finally, 56.6% of participants agreed with the statement “I found some positives in this experience” (factor 5).

**Table 1:** Participants’ socio-demographic and professional characteristics (N = 1077)

Variables	Values
Sex, % (N)	
female	71.1 (757)
male	28.9 (307)
**Age, mean ± sd (N)**	48.0 ± 10.3 (1017)
**Educational level, % (N)**	
middle school degree	2.7 (29)
high school degree	24.6 (262)
bachelor degree	31.0 (331)
master degree	41.7 (445)
**Professional role, % (N)**	
psychiatrist	21.9 (233)
psychologist	8.2 (87)
nurse	34.1 (363)
health care assistant	5.4 (57)
rehabilitation specialists, educators, other rehabilitation staff	19.4 (206)
administrative staff	1.5 (16)
social cooperative worker	3.4 (36)
social worker	4.4 (47)
peer supporter, volunteer, other	1.7 (18)
**Years of work in the mental health field, mean ± sd (N)**	16.5 ± 10.7 (1011)
**Main place of work, % (N)**	
Community Mental Health Center - CMHC	56.5 (603)
Day Center - DC	6.3 (67)
General Hospital Psychiatric Unit - GHPU	9.7 (104)
Residential Facilities - RF	12.5 (133)
Social Cooperative - SC	10.3 (110)
Other	4.7 (50)

**Table 2:** Views of MHS staff on positive changes during the first two year of the COVID-19 pandemic (total sample N = 1077)

QT-19 S items	Not really true			Really true			Missing
**Factor 1 - Acknowledgement of user capabilities**	1%	2%	3%	4%	5%	6%	N
*, people with severe mental disorder demonstrated good adaptive skills	5.0	13.0	22.1	27.0	23.3	9.6	5
*, I discovered personal resources in users that I did not believe they had	2.7	4.3	12.4	26.8	**34.0**	**19.7**	8
*, users organized themselves into peer support groups	21.3	23.5	21.1	16.3	11.6	6.2	16
*, users showed that they were able to self-organize and find new solutions	5.7	10.7	19.7	28.5	22.5	12.8	16
*, I realized that there were users who know how to use digital technologies better than I did	13.8	13.8	16.3	18.1	18.4	19.6	5
**Factor 2 -Awareness and value of teamwork**							
*, we realized the importance of simple gestures like drinking coffee together, shaking hands, hugging each other	1.6	2.3	5.3	8.8	**21.8**	**60.2**	5
*, there was more sharing of responsibilities within the team	4.0	8.1	14.1	21.1	**25.6**	**27.1**	11
*, more centrality was given to the meetings and to dialogue with the other person	2.7	6.9	14.7	25.8	27.3	22.6	4
*, I had more time to think about my work	12.4	12.6	16.2	18.5	22.1	18.2	6
*, we colleagues strengthened each other to face the fear	2.5	4.7	9.2	18.0	**26.5**	**39.1**	10
*, the sense of being part of a team strengthened	8.3	10.3	17.9	24.1	20.8	18.6	7
*, service meetings were an opportunity for group reflection on work practices	7.1	8.6	14.1	22.7	26.7	20.7	7
*, Local Health Authority’s guidelines on how to work safely made us feel more reassured	10.2	12.0	19.8	23.8	21.5	12.7	5
**Factor 3 - Flexibility and ability to reinvent the service**							
*, we became more flexible toward remaining close to users	3.0	4.8	10.8	20.4	**33.7**	**27.4**	8
* We reinvented our way of working in line with government mandates	1.4	2.5	5.7	13.1	**30.3**	**47.1**	6
*, we were able to make organizational/operational changes very quickly	2.3	3.9	8.9	20.1	**30.9**	**33.7**	4
*, we mediated between user demands and the procedures needed to work safely	0.5	1.6	3.8	13.4	**35.9**	**44.9**	7
**Factor 4 - Maintenance and introduction of best practices**							
*, we increased phone contact with users	3.1	1.8	6.2	8.9	**26.6**	**53.4**	21
*, we took more better care maintaining cleanliness in the work environment	1.0	1.4	2.4	8.7	**23.6**	**62.9**	4
*, we placed more importance on family members and the close relationships of users	1.9	4.7	12.0	21.8	**29.7**	**29.9**	10
*, we learned to use digital communication technologies to work with other public institutions and the third sector	5.3	4.9	10.1	16.8	**27.9**	**35.0**	3
*, we paid more attention to the physical health of users	1.6	4.0	11.6	22.6	**29.2**	**30.9**	4
*, we redefined the use of service spaces in a more rational way	2.3	4.2	10.1	13.6	**27.5**	**42.2**	2
*, we revised the users’ programs according to their new needs	1.2	3.7	8.7	22.7	**32.3**	**31.4**	11
*, learned to use the PC and digital technologies better (e.g., video calling and conferencing platforms)	1.5	2.3	5.0	12.7	**27.4**	**51.2**	11
*, the CMHC remained the key point of referral for people with a fragile/absent family network	4.2	4.4	9.6	15.3	**29.8**	**36.6**	28
*, during hospitalization we guaranteed the contact of users with their families	5.1	6.0	13.7	18.9	**29.2**	**27.2**	91
*, we managed people in crisis as much as possible at home	6.9	6.4	11.6	17.9	**28.2**	**28.9**	61
*, we informed users about the pandemic and how to reduce individual risk of infection	0.5	1.8	3.7	9.9	**28.0**	**56.1**	7
**Factor 5 - Acknowledgement of positive aspects in the pandemic experience**							
*, I found some positives in this experience	5.6	5.0	10.5	22.3	**29.3**	**27.3**	4

Cronbach’s alpha values: 0.72 (factor 1), 0.84 (factor 2), 0.73 (factor 3), 0.82 (factor 4). In bold: items with more than 50% of responses in scores 5 and 6.

### Differences in Staff Views Related to socio-demographic and Professional Characteristics

Compared to male staff, female staff perceived MHS as more flexible and capable to maintain best practices and female staff acknowledged more capabilities to the users (Wilks’s λ = 0.98, F (5, 997) = 5.03, p < .001; Table 3). Gender differences were influenced by work experience (Wilks’s λ = 0.98, F (5, 997) = 5.10, p < .001), particularly regarding respondent’s perception of maintenance of good practice (F = 4.53, p < .05) and acknowledgement of users’ abilities (F = 21.77, p < .001).

**Table 3:** Views of MHS staff on positive changes during the pandemic: gender differences

	Participant gender		
QT-19 S factors	Males (N = 287)	Females (N = 717)	MANOVA
	mean ± SE	mean ± SE	F (1, 1003)
Acknowledgement of user capabilities	3.67 ± 0.06	3.83 ± 0.04	5.6*
Awareness and value of teamwork	4.28 ± 0.06	4.32 ± 0.04	0.32
Flexibility and ability to reinvent the service	4.81 ± 0.05	4.97 ± 0.03	7.2**
Maintenance and introduction of best practices	4.75 ± 0.04	4.90 ± 0.03	9.4**
Acknowledgement of positive aspects in the pandemic experience	4.53 ± 0.08	4.46 ± 0.05	0.56

Covariate: years of work in the mental health field: Wilks’s λ = 0.98, F (5, 997) = 5.03, p < .0001; * <.05; ** p < .01; ***p < .0001

Significant differences were detected in respondents’ perception of MHS flexibility and capacities to maintain best practices among workplaces (Wilks’s λ = 0.87, F (20, 3158.8) = 6.91, p < .001; Table 4). At post hoc analysis, perception of flexibility was lower among staff of general health psychiatric units vs. staff of cooperatives and perception of maintenance of best practices was lower among staff of general health psychiatric units and residential facilities than among staff of community mental health centres. Differences were influenced by work experience (Wilks’s λ = 0.98, F (5, 952) = 3.90, p < .01), particularly with respect to respondents’ perception of teamwork value (F = 4.64, p < .05) and acknowledgement of users’ capacities (F = 15.47, p < .001).

**Table 4:** Views of mental health services staff on positive changes during the pandemic: mental health service differences

	Type of mental health service					
QT-19 S factors	CMHC (N = 573)	DC (N = 66)	GHPU and DH (N = 95)	RF (N = 125)	Coop (N = 103)	MANOVA
	mean ± SE	mean ± SE	mean ± SE	mean ± SE	mean ± SE	F (4,961)
Acknowledgement of user capabilities	3.80 ± 0.04	3.74 ± 0.11	3.76 ± 0.10	3.78 ± 0.08	3.80 ± 0.09	0.11
Awareness and value of teamwork	4.27 ± 0.04	4.38 ± 0.12	4.40 ± 0.10	4.41 ± 0.84	4.49 ± 0.09	1.67
Flexibility and ability to reinvent the service	4.90 ± 0.035	5.00 ± 0.10	4.73 ± 0.09^a^	4.97 ± 0.07	5.15 ± 0.08^b^	3.57**
Maintenance and introduction of best practices	4.49 ± 0.03^a^	4.93 ± 0.08	4.69 ± 0.07^b^	4.66 ± 0.06^b^	4.80 ± 0.07	6.54***
Acknowledgement of positive aspects in the pandemic experience	4.50 ± 0.06	4.48 ± 0.17	4.41 ± 0.14	4.43 ± 0.13	4.55 ± 0.14	0.18

CMHC – Community Mental Health Centers; DC – Daily Centers; GHPU – General Hospital Psychiatric Unit; DH – Day Hospitals; RF Residential Facilities; Coop – Cooperatives. Covariate: years of work in the mental health field: Wilks’s λ = 0.87, F (20, 3158.8) = 6.91, p < .0001; * <.05; ** p < .01; ***p < .0001; Bonferroni post-hoc comparisons: a < b

Respondents’ views differed between professional categories in all aspects but the perception of the pandemic as a positive experience (Wilks’s λ = 0.90, F (20, 3228.03); = 5.09, p < .0001; Table 5). Notably, psychologists had the lowest mean scores in each aspect. At post-hoc: psychologists were more skeptical on users’ capacities and teamwork value than any other professional category, and psychologists had a lower perception of maintenance of best practice than nurses; psychiatrists acknowledged less value to teamwork, compared to nurses and social health workers; psychiatrists and psychologists had lower perception of MHS flexibility vs. socio-health workers. Differences among professional categories were influenced by work experience (Wilks’s λ = 0.98, F (5, 973) = 3.85, p < .01) as far as teamwork value (F = 3.93, p < .05) and user capacities’ acknowledgment (F = 17.44, p < .001).

**Table 5:** Views of mental health services staff on positive changes during the pandemic: professional role differences

	Participants’ professional roles					
QT-19 S factors	Psychiatrists (N = 229)	Psychologists (N = 85)	Nursing staff (N = 335)	Educators, rehabilitation staff social workers (N = 248)	Health assistant, other coop staff (N = 86)	MANOVA
	mean ± se	mean ± se	mean ± se	mean ± se	mean ± se	F (4, 982)
Acknowledgement of user capabilities	3.81 ± 0.06^b^	3.29 ± 0.10^a, c^	3.87 ± 0.05^d^	3.83 ± 0.06^d^	3.73 ± 0.10^d^	6.98***
Awareness and value of teamwork	4.1 ± 0.06^a^	3.97 ± 0.10^c^	4.49 ± 0.05^b,d^	4.30 ± 0.06^d^	4.49 ± 0.10^b,d^	9.33***
Flexibility and ability to reinvent the service	4.82 ± 0.05^a^	4.69 ± 0.09^c^	4.97 ± 0.05	4.96 ± 0.05	5.17 ± 0.09^b,d^	4.98**
Maintenance and introduction of best practices	4.83 ± 0.05	4.68 ± 0.07^a^	4.97 ± 0.04^b^	4.81 ± 0.04	4.81 ± 0.08	4.01**
Acknowledgement of positive aspects in the pandemic experience	3.8 ± 0.06	3.29 ± 0.10	4.53 ± 0.08	4.51 ± 0.09	4.53 ± 0.15	0.37

Covariate: years of work in the mental health field; Bonferroni post-hoc comparisons: a < b; c < d; Wilks’s λ = 0.90, F (20, 3228.03) = 5.09, p < .0001; * <.05; ** p < .01; ***p < .0001

Finally, the three geographical areas revealed differences in staff views of teamwork value, maintenance of best practice and acknowledgement of positive aspects in the pandemic (Wilks’s λ = 0.95, F (10, 1898) = 5.22, p < .0001; Table 6). At post-hoc, staff working in southern Italy MHS: reported higher mean scores in the above-mentioned aspects than northern Italy staff; gave more values to teamwork than central Italy MHS staff. Differences among geographical areas were influenced by work experience (Wilks’s λ = 0.98, F (5, 949) = 4.35, p < .001), with respect to staff acknowledgement of users’ abilities (F = 16.33, p < .001).

**Table 6:** Views of mental health services staff on positive changes during the pandemic: MHS geographical area differences

	MHS geographical area			
QT-19 S factors	Northern Italy (N = 507)	Central Italy (N = 159)	Southern Italy (N = 291)	MANOVA
	mean ± SE	mean ± SE	mean ± SE	F (2,961)
Acknowledgement of user capabilities	3.76 ± 0.04	3.81 ± 0.08	3.85 ± 0.06	0.76
Awareness and value of teamwork	4.17 ± 0.04^a^	4.33 ± 0.08^a^	4.59 ± 0.06^b^	17.68***
Flexibility and ability to reinvent the service	4.90 ± 0.04	5.00 ± 0.07	4.99 ± 0.05	1.14
Maintenance and introduction of best practices	4.82 ± 0.03^a^	4.89 ± 0.05	4.97 ± 0.04^b^	4.55**
Acknowledgement of positive aspects in the pandemic experience	4.42 ± 0.06^a^	4.44 ± 0.11	4.69 ± 0.08^b^	3.74*

Covariate: years of work in the mental health field: Wilks’s λ = 0.95, F (10, 1898) = 5.22, p < .0001; * <.05; ** p < .01; ***p < .0001; Bonferroni post-hoc comparisons: a < b

## Discussion

This study showed that, at least limited to participants, over the two-year pandemic emergency MHS staff felt to have maintained best practices and reinvented the services, rediscovering the values of the teamwork and acknowledging unexpected capabilities in the users. The study also found that participants’ views differed with respect to gender, workplace, professional roles, and geographic area, and that these differences covaried with work experience. These results, which are consistent with those found at one year pandemic in a community-oriented MHS in Italy (MANOVA, Wilks’s λ = 0.99, F = 1.68, df 5, 1255, p = .13; Magliano et al., 2022), would suggest that MHS responsiveness continued throughout the two years of the emergency and was widespread, albeit with geographic differences, throughout the country. These results are particularly comforting considering that recent years have seen a depletion of resources allocated to MHS, which has contributed to a shift in care toward almost exclusively pharmacological interventions (Lora et al., 2022). In line with the interpretation of the results of the one-year pandemic study mentioned above (Magliano et al., 2022), the results of this study can be interpreted according to the transactional model of Lazarus and Folkman (1984). Based to this model, the adaptation to the pandemic can be schematically divided into two successive stages of cognitive appraisal. The primary appraisal encompassed the reactions and strategies implemented in the initial stages of the pandemic. The secondary appraisal concerned the emotional and problem-oriented strategies for coping with the emergency in the long term. The data reported here suggest that the interaction between internal factors, such as attitude and personal meaning of care work and experience, and external factors, such as team cohesion and MHS organization, may have influenced the adaptation process. This, in its turn, may have led MHS staff to draw a “positive message” from the pandemic experience, despite the undeniable difficulties.

From the participant perspective, the pandemic might have fostered significant changes in service practices, including increased use of home-based crisis management, greater attention to the physical health of users, and rapid digital literacy of the team. Some data corroborate those of previous studies, as those on the increased use of tele-medicine during the pandemic (Witteveen et al., 2022). The high number of items with more than 50% responses in scores 5 and 6 suggests that MHS staff managed to be resilient in the face of the adversity of two years of pandemic. The results of this study indirectly support those of a survey by Pappa et al. (2021) on the psychological well-being of MHS staff in the UK. The survey revealed that in the early phase of the pandemic, 70% of participants had high levels of resilience and 25% moderate levels. The valuing of teamwork, attested to by 82.0% of respondents who stated they realized the importance of simple gestures and by the 65.0% of respondents who reported they strengthened each other, confirms the relevance of mutual support as a key resource to deal with the pandemic stress in the workplace (McCann et al., 2013; Johnson et al., 2021). It should be also emphasized the recognition of unexpected personal resources in the users, admitted by the 54% of participants. Valuing people with mental disorders as able to cope with a complex difficulty such as the pandemic could facilitate de-stigmatization and user empowerment in mental health care settings (Magliano et al., 2017; WHO, 2010).

In this study, female staff showed higher perception of the service as able to reinvent itself and maintain best practices, and the users as capable to deal with the pandemic. These findings are in line with the results of a study on coping strategies among health professionals conducted in Italy in the early phase of the pandemic (Italia et al., 2021). In that study, female workers scored higher in effective coping strategies than male staff, particularly in the use of social support. The greater ability of women to cope with emergencies using flexible coping strategies (Italia et al., 2021) may in turn explain the greater involvement of women in the health professions (Lotta et al., 2021; Ministero della Salute, 2013). The more positive perceptions about changes in MHS during the pandemic found among female vs. male participants are consistent with findings of McCann et al. (2013) about gender differences in the resilience of healthcare workers in the face of work stress. Given the paucity of studies specifically addressing gender differences in professional reactions to pandemic (Dragioti et al., 2022; Mastroberardino et al., 2022; Pappa et al., 2021), further research on this topic is advisable.

The study also revealed that perceptions of flexibility and maintenance of best practices were higher among staff working in community services than among those working in hospital/residential facilities. These findings, which emphasize the responsiveness of community services to pandemic (Johnson et al., 2021) could be partly related to the support provided by user and family associations to community services (Fioritti et al., 2021) and the centrality of community services in Italy (WHO, 2021). Conversely, the clinical conditions of users and the characteristics of inpatient facilities may have resulted in lower perceptions of positive transformation among the staff of these facilities (De Girolamo et al., 2020). The increased workload in inpatient facilities (in some cases with the temporary shift of staff from psychiatric units to physical medicine wards) and the higher risk of contagion compared to outpatient facilities (Foye et al., 2021) should also be considered.

The pandemic emergency appears to have been a transformative opportunity especially for non-clinical professionals (Itzhaki-Braun, 2021). This could be partially explained by the fact that during the pandemic professionals as rehabilitators and nurses took a more active role in case management, mobilizing underused professional competencies. In contrast, psychologists and psychiatrists have had to manage more clinical responsibilities and a heavier workload due to increased care needs from other health professionals and the general population. Furthermore, psychiatrists and psychologists may have had to limit their previous relational activities with users (e.g., clinical talks and psychotherapeutic sessions) experiencing a sense of helplessness and isolation.

Apparently surprising is the greater perception of maintenance of best practices, teamwork valuing, and acknowledgement of positive aspects from the staff of southern Italy, a geographic area where health resources are the poorest in the country (ISTAT, 2022c; Lora et al., 2022). Differences in favor of southern Italy may depend on factors as the lower mortality from COVID-19, mainly in the first pandemic waves (ISTAT, 2022a; Ministero della Salute, 2022a) and the lower number of large residential facilities (Istituto Superiore di Sanità, 2022; ISTAT, 2022b) which may have led to the need for fewer restrictions. Greater perception of positive transformation among the staff from Southern Italy MHS vs. those from Central and Northern MHS might be also related to cultural characteristics. In the pandemic period, the social indicator “satisfaction with social relationships” decreased less in southern Italy than in central and northern areas (ISTAT, 2022c). The greater social tenure of southern Italy may have facilitated the maintenance of best practices through the development of innovative solutions and the co-sharing of workload. Another explanation may be related to the health professionals’ coping strategies. In a study by Italia et al. (2021), the most frequently adopted strategies by health professionals in southern Italy were positive attitudes toward the workplace, namely Activation, Planning, and Acceptance. The study’s authors argued that in this geographical area, higher workloads and more demanding tasks corresponded to more significant planning activities. These, in turn, may have led the staff to acknowledge more positive transformations related to COVID-19 management. Finally, it should also be mentioned the greater relevance of the religion in southern Italy (Italia et al., 2021; Magliano & Affuso, 2022), which may have function as emotion-focused coping strategy, particularly in the early pandemic stages.

This is the first national study conducted in Italy that has specifically examined the MHS transformations over the two-year pandemic emergency from the perspective of the staff. It is noteworthy that the study specifically examines responsiveness, a dimension of Health Services Quality Assessment also including aspects of staff satisfaction (Murray & Evans 2003 cited by Morosini, 2004). These aspects are difficult to investigate retrospectively, both because of the time bias and because national information systems (in Italy, the SISM) are mainly centered on monitoring indicators of management quality and volume of services delivered. The low percentage of missing responses in most items suggests that the questionnaire captured aspects that participants felt to be relevant to their professional experience during the pandemic emergency. The use of a validated, online instrument may facilitate the replication of the survey in other mental health and non-mental health settings (such as child neuropsychiatry services and maternal and child health services) and comparisons over time.

The study has relevant methodological weaknesses to be considered when interpreting the results. These limitations include: the representativeness of the enrolled MHS. The 17 participating MHS corresponded to 40.5% of MHS whose Director was sent the initial invitation email and to 12.8% of MHS in Italy. It is likely that the Departments more severely hit by the pandemic did not accept to be enrolled, just because of the enduring stress of the staff; the representativeness of the participating staff. Although the sample size is quite large, it does not fully reflect the distribution of professional roles in the Italian MHS. Data from the National Mental Health Information System (Ministero della Salute, 2022b) report that the total staffing of public psychiatric units amounts to 29,785 workers. Of these, 17.9% are physicians, 6.9% are psychologists, 42.9% are nurses, 11.6% are healthcare assistants and 13.9% are educators, psychiatric rehabilitation technicians and social workers. Comparison of the national data with those of the study sample for the five professional categories examined in the multivariate analyses (27,417 vs. 993) shows that the sample is not fully representative (χ^2^ = 158.06, df 4, p < .0001); low staff participation in some MHS (fewer than 30 participants). This could be due to an inaccurate study dissemination strategy. Respondents are likely to be the most motivated and cooperative staff members, so their opinions may be quite different from those who declined to participate. Moreover, it cannot be excluded that poor participation was due to staff workload and conflicts between colleagues caused by the pandemic situation (LaSalvia et al., 2020); the instrument tone. The QT-19 S asked only about positive consequences of the COVID pandemic. This may have led to an imbalance in responses; the instrument’s contents. The questionnaire did not inquire about the staff’s perception or observation of the patient outcome, nor the effect of the pandemic on the user outcome. Therefore, it is not possible to verify directly whether the staff perspective fit the general reality of the patients to some degree. Some studies on the effects of the pandemic on mental disorders have shown clinical worsening, while others have revealed an association between reduced social relationships and increased empathy toward people with severe mental disorder (Gillard et al., 2021). In some cases, attention to the pandemic seems to have distracted some users from their mental conditions, leading to some degree to symptom relief (Sheridan Rains et al., 2021); the level of staff’s digital literacy. There might be a bias in responses related to the level of digital competence of staff and facilities. This bias may have been partly overcome by the fact that the instrument was multiple-choice (click-selectable) and fillable via one’s smartphone; the risk of violation of anonymity. The information collected may have involved a relative lack of anonymity, which may have been partially limited by leaving all questions unobligated; the focus only on the *perception* of positive transformations. Therefore, it is not possible to demonstrate whether the changes perceived by staff reflected the reality of MHS practices during the pandemic; the lack of qualitative data. Data from the two QT-19 additional open-ended items (response rate: 66%) will be analyzed specifically in their opposite positive/negative qualities later, by accounting for the professional role of the respondents and the type of mental health service (hospital/residential or community).

Even with the limitations mentioned above, this study provides insights into how socio-demographic variables, professional role, and workplace may have contributed to the responsiveness of Italian MHS in the first two years of the pandemic. The results of this study show, without denying the enormous difficulties faced by the MHS during the pandemic, that at least for some MHS professionals, the COVID experience was also an opportunity to rediscover the values inherent in mental health care work in the community. We hope that these findings may be useful for planning community-oriented MHS in the post-pandemic period, considering both the experience gained by staff and the process of adaptation of the MHS occurred over the COVID-19 emergency. To achieve this goal, there is an urgent need to invest substantially in human and economic resources for MHS to have skilled, capable, and motivated MHS workforce (Ministero della Salute, 2022c).

## Data Availability

The data and material (questionnaire) that support the findings of this study are available from the corresponding author upon reasonable request, which must include a protocol and statistical analysis plan and not be in conflict with our publication plan.

## Declarations

### Standards of reporting

The manuscript was prepared in accordance with STROBE guideline and checklist for observational studies as reported in the EQUATOR Network https://t.ly/kbsB.
